# A Laugh Producing Plant

**Published:** 1891-11

**Authors:** 


### A LAUGH PRODUCING PLANT.

While the Stanley expedition was crossing a portion of the southern extremity of the Sahara Desert, they were made acquainted with the peculiar properties of a plant known only to that region, called by the Arabs cuili koia, or the laughing plant. Professor Salchi, attached to the expedition, was fortunate to procure several fine specimens of this peculiar plant, which he is at present cultivating, with a view to practical experiments.

The production of laughter by artificial means, it is thought, can be reduced to a science, now that the discovery of a plant, the properties of which are a direct incentive to laughter, has been made. Any amount of cachinnation can be produced by simply increasing or diminishing the laugh producing dose.

There was a time when the somnolent effects produced by the poppy were not generally known, but the soporific properties of this plant are now beyond cavil, and in a short time it is expected that Professor Salchi will have a crop of the laugh-producing plants large enough for practical experiments. The now almost unknown plant will soon become a staple article of commerce, and the principal cereal cultivated in many a vast garden will be the laughter producing plant. As opium is certain to produce sleep, so can the laughter plant be at all times relied upon to produce laughter in all animal creatures from the micro-organisms of the oscillatoriæ up to the genus homo.

This strange plant grows in the arid deserts of Arabia, and on the vast sea of the white sand known as the Desert of Sahara, in Africa. The plant is of moderate size, with bright yellow flowers and soft, velvety seed pods, each of which contains two or three seeds, resembling small black beans. The natives of the district where this strange plant grows dry the seeds in the sun and reduce them to a fine impalpable powder by a process of maceration between two stones. A small dose of this powder has similar effects to that arising from the inhalation of laughing gas. It causes the most sober person to dance, shout and laugh with the boisterous excitement of a madman, and to rush about, cutting the most ridiculous capers for about an hour. At the expiration of this time exhaustion sets in, and the excited person falls asleep, to awake after an hour or more with a more or less vivid recollection of having been in the seventh heaven of enjoyment.

